# Suit for Mal-practice

**Published:** 1846-10

**Authors:** 


*Suit for Mal-practice.*—Another case of prosecution for mal-practice has been reported in the Boston Medical Journal, in which (the parties residing in New York) the defendant was triumphantlyacquitted. The case was a comminuted fracture of the femur near the condyles, in a man of bad constitution, aged 64.—The result was that the leg was shortened two, or two and a half inches. The defendant was sustained by the testimony of Drs. Parker, Reese, Post, Wood, Mott, and Cheesman. Some parts of the testimony of these men deserves to be recorded, as remarkable for its clearness and *truth*—"truth," we say, because there are not a few surgeons of less experience and reputation than either of the above surgeons, who never, if their assertions are to be believed, make shortened limbs; and who consequently are always prepared to bear testimony against their less fortunate neighbours.

Says Dr. Reese, "there could be no doubt that it was an oblique and comminuted fracture, which is always unfavourable *and renders a shortening of the limb inevitable.*

Says Dr. Post. "In *all* such cases a very considerable shortening of the limb takes placd under the best treatment."

Dr. Cheesman "found the limb shorter than the other, as it uniformly is in such cases. *He never knew an exception.*"

Dr. Mott testified that "more or less shortening of the limb is the *result after fractured thigh, even in the most favourable circumstances.*"

Prof. Parker, thought "the patient was exceedingly well off to have recovered from such an accident with both his life and limb, and with no other disease than a shortened leg.—*Buffalo Med. Journ.*

---

Dr. George Chandler has been appointed Superintendent of the Mass. State Lunatic Asylum, at Worcester, Dr. Woodward having resigned the office. Dr. C. was formerly superintendent of the Insane Asylum, at Concord, New Hampshire. A committee of the citizens of Worcester has been appointed to procure a marble bust of Dr. Woodward, to be placed in the hospital.—*Ib!*

